# Endogenous zinc depresses GABAergic transmission via T-type Ca^2+^ channels and broadens the time window for integration of glutamatergic inputs in dentate granule cells

**DOI:** 10.1113/jphysiol.2013.261420

**Published:** 2013-09-30

**Authors:** Antonia Grauert, Dominique Engel, Arnaud J Ruiz

**Affiliations:** 1 Department of Pharmacology, School of Pharmacy, University College LondonLondon, UK; 2 Giga-Neurosciences, University of Liège, LiègeBelgium

## Abstract

**Abstract **Zinc actions on synaptic transmission span the modulation of neurotransmitter receptors, transporters, activation of intracellular cascades and alterations in gene expression. Whether and how zinc affects inhibitory synaptic signalling in the dentate gyrus remains largely unexplored. We found that mono- and di-synaptic GABAergic inputs onto dentate granule cells were reversibly depressed by exogenous zinc application and enhanced by zinc chelation. Blocking T-type Ca^2+^ channels prevented the effect of zinc chelation. When recording from dentate fast-spiking interneurones, zinc chelation facilitated T-type Ca^2+^ currents, increased action potential half-width and decreased spike threshold. It also increased the offset of the input–output relation in a manner consistent with enhanced excitability. In granule cells, chelation of zinc reduced the time window for the integration of glutamatergic inputs originating from perforant path synapses, resulting in reduced spike transfer. Thus, zinc-mediated modulation of dentate interneurone excitability and GABA release regulates information flow to local targets and hippocampal networks.

**Key points**

- Zinc inhibits ionotropic receptors commonly found at central synapses, as well as a wide variety of voltage-activated ion channels that modulate neuronal excitability and neurotransmitter release.
- We found that zinc chelation facilitated GABAergic signalling in dentate granule cells and that blocking T-type Ca^2+^ channel activity abolished this effect. Zinc chelation reduced spike threshold, increased spike width and shifted the input–output relationship in dentate interneurones, which is consistent with increased excitability.
- In granule cells, zinc chelation narrowed the window for the integration of glutamatergic inputs originating from perforant path synapses.
- These results demonstrate that zinc modulates dentate interneurone function and regulates spike routing to local and hippocampal targets.

The vesicles of many glutamatergic terminals in the mammalian forebrain are heavily enriched in ionic zinc, the function of which is poorly understood. Recent studies imply that zinc is released upon presynaptic activity, and diffuses and binds to both pre- and postsynaptic receptors, where it acts as a modulator of cellular excitability and synaptic plasticity (for reviews, see Smart *et al.* 2004; Paoletti *et al.* 2009; Toth, 2011). Among the roles proposed for endogenous zinc are the triggering of apoptotic pathways (Aizenman *et al.* 2000), regulation of gene expression (Tsuda *et al.* 1997) and epileptogenesis (Buhl *et al.* 1996), progression of Alzheimer’s disease (Duce *et al.* 2010) and altered pain signalling (Nozaki *et al.* 2011). Zinc directly inhibits a number of ionotropic receptors commonly found at central synapses including NMDA (Paoletti *et al.* 2000; Gielen *et al.* 2009), GABA_A_ (Barberis *et al.* 2000; Hosie *et al.* 2003; Ruiz *et al.* 2004), glycine (Mayer & Vyklicky, 1989) and kainate receptors (Mott *et al.* 2008; Veran *et al.* 2012). It also inhibits glutamate transporters (Spiridon *et al.* 1998) and the neuronal K^+^-Cl^−^ co-transporter KCC2 (Hershfinkel *et al.* 2009), as well as a wide variety of voltage-activated channels that are important for the fine-tuning of cellular excitability and neurotransmitter release. For example, zinc efficiently inhibits T-type Ca^2+^ channels containing the Ca_v_3.2 subunit (Sun *et al.* 2007; Traboulsie *et al.* 2007) or K^+^ channels containing the K_v_3.1 subunit (Gu *et al.* 2013) or the K_v_1.1 subunit (Imbrici *et al.* 2007). Finally, it potently inhibits GABA_A_ receptors lacking the γ-subunit (Smart *et al.* 1991). Despite this wealth of evidence, and the demonstration that endogenous zinc can affect glutamatergic transmission in the hippocampus (Vogt *et al.* 2000; Molnar & Nadler, 2001a), the amygdala (Kodirov *et al.* 2006) and the retina (Wu *et al.* 1993), it remains unclear how it may alter the functionality of GABAergic networks. Hitherto, studies undertaken in the hippocampal formation have mainly focused on monosynaptic inputs (Vogt *et al.* 2000; Ruiz *et al.* 2004; Huang *et al.* 2008) and there has as yet been little attempt to dissect how zinc modulates di-synaptic events, particularly those mediated by GABA_A_ receptors. Furthermore, very few studies have provided a detailed characterization of the actions of zinc in dentate interneurones (Koh *et al.* 1995; Berger *et al.* 1998) and none have investigated how zinc signals might modulate the window of integration of excitatory synaptic inputs and spike transfer in principal cells.

Zinc is extremely abundant in the axon of dentate granule cells (or mossy fibres), which ramify extensively and make contact ‘en passant’ or via filopodia to a large variety of interneurones in both the hilus and stratum lucidum (Freund & Buzsaki, 1996; Szabadics & Soltesz, 2009). Morphological and electrophysiological analysis of GABAergic interneurones in the dentate gyrus revealed fast-spiking, parvalbumin-expressing cells that preferentially innervate the somatic and perisomatic regions of granule cells, and are essential for the generation of gamma oscillations (Bartos *et al.* 2007, 2011). Other classes of interneurones in the dentate gyrus with axons that are confined more distally to the termination zone of perforant path inputs include molecular layer-associated interneurones (MOPP) and hilar perforant path-associated interneurones (HICAP). Mossy fibre-associated interneurones that are found in stratum lucidum largely contribute to feed-forward inhibition in CA3 pyramidal neurones (Buzsaki, 1984; Acsady *et al.* 1998), whereas hilar and dentate interneurones innervated by recurrent mossy fibre collaterals provide a feedback input that regulates dentate granule activity (Penttonen *et al.* 1997; Doherty *et al.* 2004; Ewell & Jones, 2010; Sambandan *et al.* 2010). Electrical stimuli designed to release zinc at this recurrent mossy fibre pathway did not affect GABA_A_ receptor-mediated currents evoked in granule cells by photo-uncaging GABA (Molnar & Nadler, 2001b). This result does not, however, exclude the possibility that zinc may alter GABAergic signalling in granule cells by modulating interneurone activity and thus di-synaptic inhibition.

We demonstrate here that zinc depresses GABAergic transmission to granule cells and that this modulation occurs via T-type Ca^2+^ channels. We also show that zinc chelation selectively broadens the action potential waveform in dentate fast-spiking interneurones and that it narrows the window for the integration of glutamatergic inputs originating from perforant path synapses. Our results unravel a phenomenon whereby zinc depresses interneurone excitability and modulates spike routing to the hippocampus proper.

## Methods

### Hippocampal slice preparation

All animal procedures strictly followed University College London (UCL) Research Ethics Committee regulations. Sprague–Dawley rats (Harlan Laboratories Ltd, Oxon, UK) aged 20–40 days were killed by overdose of sodium pentobarbital injected intraperitoneally (100 mg kg^−1^) and rapidly decapitated in accordance with the UK Animals (Scientific Procedures) Act, 1986. Transverse 250–300 μm thick slices were obtained from both hippocampi using a vibratome [Leica VT-1200S; Leica Biosystems (UK) Ltd, Milton Keynes, UK]. Slices were kept at 35°C for 30 min after slicing and then at room temperature (22°C). For the dissection and storage of slices, the solution contained NaCl (87 mm), NaHCO_3_ (25 mm), d-glucose (10 mm), sucrose (75 mm), KCl (2.5 mm), NaH_2_PO_4_ (1.25 mm), CaCl_2_ (0.5 mm) and MgCl_2_ (7 mm). For experiments, the slices were superfused with physiological saline containing NaCl (125 mm), NaHCO_3_ (25 mm), d-glucose (25 mm), KCl (2.5 mm), NaH_2_PO_4_ (1.25 mm), CaCl_2_ (2 mm) and MgCl_2_ (1 mm), equilibrated with 95% O_2_/5% CO_2_. All solutions were prepared using water purchased from Fisher Scientific UK Ltd (Loughborough, UK).

### Electrophysiological recordings

Patch pipettes (3–5 MΩ) were pulled from borosilicate glass (1.5 mm outer diameter, 0.5 mm wall thickness) and recordings were obtained from granule cells and dentate interneurones under infrared differential interference contrast imaging at 22°C. We placed bipolar stainless steel stimulating electrodes in stratum lucidum of CA3b and stratum granulosum of the dentate gyrus. Synaptic currents were recorded with an Axopatch 200-B amplifier (Molecular Devices LLC, Sunnyvale, CA, USA), filtered at 2 kHz (internal 4-pole low-pass Bessel filter), and sampled at 10 kHz. Data were acquired and analysed offline using the Labview software environment (National Instruments Corp., Austin, TX, USA). Access resistance was monitored throughout the experiments and was <20 MΩ; results were discarded if it changed by >20%. Junction potentials were not corrected. The pipette solution used for IPSCs recorded at a holding potential (*V*_holding_) of −70 mV contained CsCl (120 mm), QX314 Br (5 mm), NaCl (8 mm), MgCl2 (0.2 mm), Hepes (10 mm), EGTA (2 mm), MgATP (2 mm), Na_3_GTP (0.3 mm) (pH 7.2, osmolarity 310 mOsm l^−1^). IPSCs evoked by stratum lucidum stimulation (20 μs, 20–100 μV) was analysed only if currents were reversibly depressed by >40% by (2*S*,2′*R*,3′*R*)-2-(2′,3′-dicarboxycyclopropyl)glycine (DCG-IV; 1 μm) consistent with the selective sensitivity of mossy fibre synapses to group II metabotropic receptor agonists. To measure IPSCs recorded near the reversal potential for glutamate receptors in granule cells (*V*_holding_= 0 mV), CsCl was substituted with Cs-gluconate. To record T-type Ca^2+^ currents, the pipette solution contained K-gluconate (130 mm), Hepes (20 mm), NaCl (8 mm), EGTA (0.2 mm), QX-314 Br (5 mm), MgATP (2 mm) and Na_3_GTP (0.3 mm). The perfusion medium contained TEA (5 mm), 4-AP (2 mm) and TTX (1 μm) to block K^+^ and Na^+^ channels and nifedipine (20 μm) to block L-type voltage-gated Ca^2+^ channels. Slices were maintained within an interface chamber on a Petri dish containing ω-agatoxin IVA (0.5 μm), ω-conotoxin GVIA (2 μm) and SNX-482 (0.5 μm) to block P/Q-, N- and R-type voltage-gated Ca^2+^ channels, respectively, for at least 2 h prior to recording. GABA_A_ and glutamate receptors were blocked by the addition of bicuculline methiodide (10 μm) and kynurenic acid (2 mm). *V*_holding_ was set to −70 mV and a 500 ms pre-pulse to −90 mV followed by a 1 s depolarizing step was applied from −90 mV to +40 mV, with a 10 mV increment. Series resistance was compensated (range: 11–23 MΩ; voltage clamp 60–70% correction, time lag 10 μs). The solution for recording from granule cell somata and fast-spiking interneurones in current-clamp mode contained K-methanesulfonate (145 mm), EGTA (2 mm), Hepes (10 mm), NaCl (5 mm), MgCl_2_ (2 mm), Na_2_ATP (2 mm), Na_3_GTP (0.5 mm) and Na-phosphocreatine (5 mm). For each neurone, the *I*–*V* relation was determined by measuring the amplitude of steady state voltage deflections elicited by a series of hyperpolarizing and depolarizing current steps. The input resistance was obtained by fitting the linear portion of the *I*–*V* relation. ‘Sag’ ratios were determined as the ratio between the steady state voltage and peak voltage in response to a current injection that resulted in a membrane potential negative to −120 mV. To determine the membrane time constant (*τ*_m_), averaged deflections of hyperpolarizing potentials (less than −10 mV) were fitted by a mono-exponential function. Maximum firing rates were determined by the interval between the first and second action potentials at a current step that did not inactivate Na^+^ channels. Action potential amplitudes were measured from *V*_m_ preceding the current pulse. The half-spike width was determined as the duration at half-spike amplitude and was calculated offline. To determine the action potential threshold, phase plots were constructed by plotting the first derivative of the membrane potential *versus* the membrane voltage (Bean, 2007). Local pressure application of l-glutamic acid monosodium salt (Glu; 100 μm in control perfusion solution) was delivered via a patch pipette connected to a Picospritzer (10–50 ms, 5–30 p.s.i.; General Valve Corp., Fairfield, NJ, USA). The pipette was first positioned in stratum lucidum in the vicinity of the stimulating electrode; after a series of puffs, it was moved to stratum granulosum at <300 μm from the other stimulating electrode. The bath perfusion was arranged to keep the granule cell body recorded upstream of this position. To calculate the frequency of Glu-evoked IPSCs, inter-event intervals were measured just after the onset of the puff and the resulting frequency subtracted from that of spontaneously occurring IPSCs without glutamate puff. Spontaneous IPSCs were identified as events exceeding the threshold of three times the standard deviation of the noise level for 80 s, in the absence of glutamate puff. To analyse the offset and gain in dentate neurones, the mean firing frequency was plotted against the injected current, thus yielding an input–output (I–O) relation for each cell. The I–O relation was then fitted over a range of firing frequencies with equations describing a logarithmic function represented by the slope of the I–O relation over the whole range of curve fitting (gain) and the *x*-offset (e.g. the amount of current required to bring the cell to fire). Control I–O relationships were subject to non-linear fitting using:


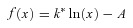
1

where *f*= firing frequency (Hz), *x*= injected current (pA), *k*= gain (Hz pA^−1^) and exp *A*/*k*= (*x*-offset). Fitting parameters *k* and *A* were then used to constrain the fitting of the I–O relationship during chelation of zinc, using:


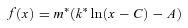
2

where the gain is equal to m*k, and *C* is the shift in offset (*Δ*_offset_) (pA). For spike-timing experiments, one stimulus electrode was positioned in stratum lucidum to activate mossy fibres and the other in the outer molecular layer >300 μm away from the recorded granule cell soma to activate the lateral perforant path. Both pathways were activated at different time intervals (from −30 ms to +30 ms, in 10 ms increments) for 10 cycles, in a control condition and after chelation of zinc. Spike probability and the amplitude of subthreshold potentials evoked by single shocks were analysed offline in responses acquired in separate channels.

### Immunohistochemistry and confocal microscopy

To recover the morphology of recorded cells, slices were transferred into 4% paraformaldehyde fixative and stored at 4°C overnight. The fixative was exchanged with PBS and cells were permeabilized in 0.5% Triton (Tx-100). Subsequently, the slices were stored in 0.1% streptavidin (Alexa 488; Invitrogen, Inc., Carlsbad, CA, USA) for 2 h. Slices were mounted on a microscope slide in DABCO-Mowiol® and allowed to dry overnight. High-resolution single-photon images were acquired at 488 nm with a Zeiss LSM 710 confocal microscope (Carl Zeiss Microscopy GmbH, Jena, Germany) equipped with a 63× oil immersion objective (1.4 numerical aperture). Anatomical 3-D reconstructions were obtained from stacks of 51–171 images per cell (voxel size: 271–758 nm in the *x*–*y* plane, 1 μm along the *z*-axis). Image stacks belonging to one cell were imported into Neuromantic Version 1.6.3 for 3-D analysis (Myatt *et al.* 2012).

### Drugs

*N*,*N*,*N*′*N*′-tetrakis(−)[2-pyridylmethyl]-ethylenediamine (TPEN), ethylenediamine tetra-acetate (EDTA), tetra-ethylamonium (TEA), 4-aminopyridine (4-AP) were obtained from Sigma-Aldrich Corp. (St Louis, MO, USA). 3-[[(3,4-Dichlorophenyl)methyl]aminopropyl]-diethoxy-methyl-phosphinic acid (CGP-52432), 7-(hydroxyim-ino)cyclopropa-[*b*]chromen-1a-carboxylate ethyl ester (CPCCOEt), (1*S*,2*S*)-2-[2-[[3-(1*H*-benzimidazol-2-yl)propyl]methylamino]ethyl]-6-fluoro-1,2,3,4-tetrahydro-1-(1-methylethyl)-2-naphthalenyl cyclopropanecarboxy-late dihydrochloride (NNC 55-0396), DCG-IV and mibe-fradil were obtained from Tocris Cookson (Bristol, UK). 2,3-Dioxo-6-nitro-1,2,3,4-tetrahydrobenzo[*f*]quinoxali-ne-7-sulfonamide disodium salt (NBQX), d-(−)-2-amino-5-phosphonopentanoic acid (d-AP5), kynurenic acid, bicuculline methiodide, TTX, and all Ca^2+^ channel antagonists were purchased from Abcam Plc (Cambridge, UK).

### Statistical tests

The results are presented as the mean ± standard error of the mean (s.e.m.). Statistical differences were determined by two-tailed Student’s *t* test for two-group comparisons unless otherwise indicated. Data were considered significant if *P* < 0.05.

## Results

### Mono- and di-synaptic GABAergic signalling in granule cells

**Figure 1:**
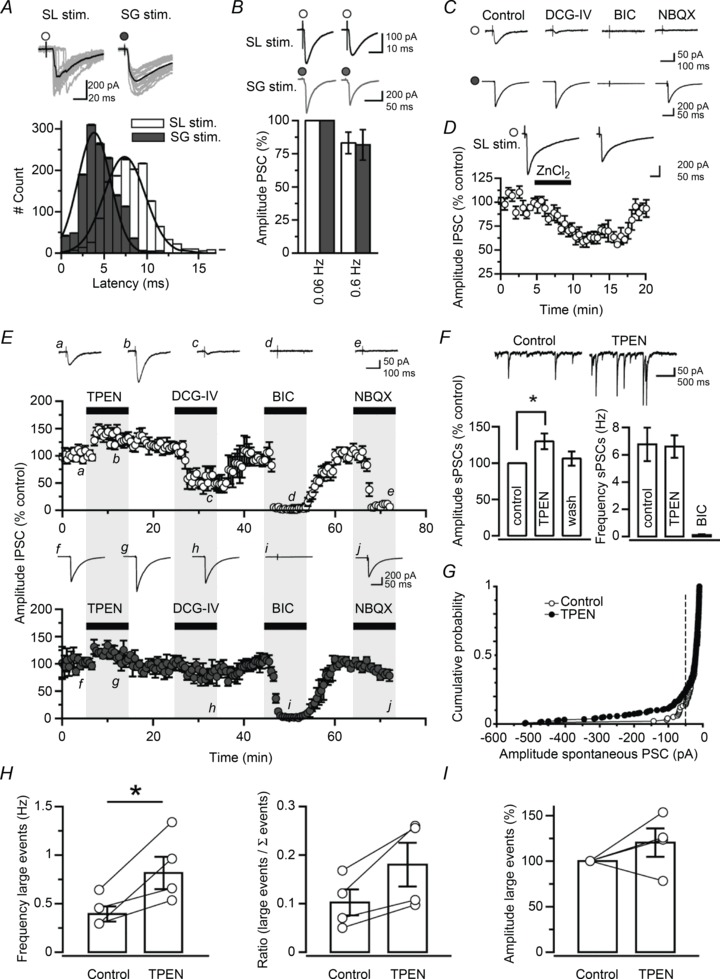
Chelating zinc with TPEN facilitates GABAergic IPSCs in granule cells *A*, distributions of latencies of PSCs evoked by stimuli delivered in stratum lucidum of the CA3b region (white) or in stratum granulosum near the tip of the ‘V’ shape formed by the layer (grey). Distributions were fitted with two Gaussian curves (data from 15 neurones). Sample recordings show 20 consecutive PSCs superimposed (grey) as well as the mean PSC (black). *B*, switching the stimulation frequency from 0.06 Hz to 0.6 Hz depresses evoked PSCs (*n*= 15). *C*, example traces from one granule cell recording (averages of 10 consecutive trials) showing the selective depression of stratum lucidum evoked PSCs (top, white circle) by the group II metabotropic glutamate receptor agonist DCG-IV (1 μm) and suppression by the AMPA and kainate receptor antagonist NBQX (20 μm) or the GABA_A_ receptor antagonist bicuculline methiodide (10 μm). Stratum granulosum evoked PSCs (bottom, black circle) are abolished by bicuculline methiodide (10 μm), but are not affected by NBQX or DCG-IV (1 μm). *D*, PSC amplitude plotted against time showing a reversible depression after the addition of zinc chloride (10 μm) to the perfusion medium (stratum lucidum pathway only, *n*= 5; *P* < 0.05, paired *t* test). *E*, plots of IPSC amplitude against time showing the effects of zinc chelation and successive pharmacological manipulations (data pooled from 20 neurones; stratum lucidum stimulation in CA3b: top, white circles; stratum granulosum stimulation: bottom, grey circles). Superfusion of TPEN (1 μm) increases IPSC amplitude at both pathways, whereas DCG-IV (1 μm) selectively inhibits IPSCs elicited by stratum lucidum stimuli. Evoked IPSCs are abolished by the addition of bicuculline methiodide (10 μm), whereas NBQX (20 μm) only suppresses stratum lucidum evoked IPSCs. Example traces for stratum lucidum (*a*–*e*) and stratum granulosum stimulation (*f*–*j*) are shown at time indicated. Grey shaded areas indicate that the effects of the drugs were monitored in parallel, at both pathways. *F*, sample traces showing spontaneous activity in a granule cell before and after superfusion of TPEN (1 μm). Note the presence of large spontaneous events. Bar graphs summarize the effects of GABA_A_ receptor blockade and zinc chelation on spontaneous PSC amplitude and frequency. Data presented from six neurones (*P < 0.05, paired *t* test). *G*, example of cumulative frequency distribution of amplitude of spontaneous PSCs in one granule cell, in control condition (white symbols) and following application of TPEN (1 μm; black symbols). The vertical dashed line indicates the threshold used for detection of large events (>50 pA). *H*, *I*, bar graphs summarize the effects of TPEN on the frequency and amplitude of large spontaneous PSCs in four neurones (*P < 0.05, paired *t* test).

We recorded PSCs (postsynaptic currents) in granule cells held in voltage-clamp in acute hippocampal slices via a patch pipette containing a high Cl^−^ concentration (*V*_holding_=−70 mV; *E*_Cl_=−1 mV). We evoked PSCs via two tungsten electrodes positioned in stratum lucidum of the CA3b region and in stratum granulosum, and routinely blocked GABA_B_ receptors and group I metabotropic glutamate receptors with CGP52432 (5 μm) and CPCCOEt (10 μm), respectively. AMPA, kainate and NMDA receptors were left unblocked to enable polysynaptic recruitment of interneurones. Evoked PSCs contained polysynaptic components with variable latencies (mean ±s.e.m. stratum lucidum stimulation: 7.39 ± 0.06 ms; mean ±s.e.m. stratum granulosum stimulation: 3.85 ± 0.02 ms; *n*= 15 cells) and were partially depressed upon switching the stimulus frequency from 0.06 Hz to 0.6 Hz (Fig. 1*A* and *B*). Superfusion of DCG-IV (1 μm) reversibly depressed stratum lucidum evoked PSCs by 44.3 ± 2.9% (*n*= 18; *P*< 0.001) but only minimally affected responses elicited by stratum granulosum stimulation (11.5 ± 2.9% amplitude depression, *n*= 18; *P* 0.1) consistent with the high sensitivity of mossy fibre synapses to group II metabotropic receptor agonists (Mann–Whitney *U*test between pathways, *P* < 0.003) and in good agreement with Doherty *et al.* ()^2004^ (Fig. 1*C*). Bath application of the GABA_A_ receptor antagonist bicuculline methiodide (10 μm) abolished evoked responses at both pathways (stratum lucidum stimulation: 98.7 ± 0.4% PSC amplitude decrease; stratum granulosum stimulation: 98 ± 0.3% decrease, *n*= 8; *P* < 0.001), whereas the AMPA and kainate receptor antagonist NBQX (20 μm) selectively depressed stratum lucidum evoked responses (88.6 ± 2.6% PSC amplitude reduction, *n*= 8; *P* < 0.001) without affecting those elicited by stratum granulosum stimulation (19.3 ± 3.2% amplitude reduction, *n*= 8; *P* > 0.1). In addition, superfusion of zinc chloride (10 μm) depressed stratum lucidum evoked PSCs by 37.9 ± 3.2% (*n*= 5; *P* < 0.05) (Fig. 1*D*). These results are consistent with di- and monosynaptic GABAergic signalling mediated by mossy fibre–interneurone–granule cell synapses and interneurone–granule cell synapses, respectively. (PSCs are named IPSCs hereafter in view of the differential sensitivity of evoked responses to NBQX and bicuculline methiodide.)

### Zinc chelation enhances GABAergic transmission to granule cells

We first asked whether zinc chelators could modulate the strength of GABAergic transmission in granule cells. Figure 1*E* shows that superfusion of the zinc chelator TPEN (1 μm), which has a femtomolar affinity for zinc, enhanced the amplitude of IPSCs evoked at both pathways (stratum lucidum stimulation: 31.6 ± 5.2%, *n*= 20; *P* < 0.05; stratum granulosum stimulation: 23.4 ± 5.1%, *n*= 8; *P* < 0.05). Subsequent application of DCG-IV (1 μm) selectively depressed stratum lucidum evoked IPSCs by 56.6 ± 7.6% (*n*= 8; *P* < 0.001), but had no effect on stratum granulosum evoked responses (8.9 ± 5.6% IPSC amplitude decrease, *n*= 8; *P* > 0.1), whereas bicuculline (10 μm) reversibly abolished both responses (*n*= 8; *P* < 0.001). Application of NBQX (10 μm) at the end of the experiment abolished stratum lucidum evoked IPSCs (*n*= 8; *P* < 0.05), but had no effect on responses elicited by stimuli delivered in stratum granulosum. When recording from granule cells held near the reversal potential for glutamate receptors (*V*_holding_= 0 mV), superfusion of TPEN increased the amplitude of stratum lucidum evoked IPSCs by 37.8 ± 6.9% (*n*= 4; *P* < 0.01). TPEN and ZnCl_2_ had little effect on the holding current measured in granule cells (Δ*I*_holding_ TPEN: 1.2 ± 2.6 pA; Δ*I*_holding_ ZnCl_2_: 6.9 ± 5.3 pA; *P* > 0.05), the fast decay-time constant of stratum lucidum evoked IPSCs (Δτ_TPEN_: 6.2 ± 2.3 ms; Δτ_ZnCl2_: 1.7 ± 1.6 ms; *P* > 0.05) or that of stratum granulosum evoked IPSCs (Δτ_TPEN_: 0.5 ± 1.4 ms; *P* > 0.7). We also analysed the effect of zinc chelation on network-driven activity in granule cells. Bicuculline (10 μm) depressed the amplitude of spontaneous events by 78 ± 9.1% and reduced their frequency from 6.8 ± 0.2 Hz to 0.1 ± 0.1 Hz (*n*= 6; *P* < 0.001), indicating that they were largely mediated by GABA_A_ receptors. Superfusion of TPEN (1 μm) reversibly increased the amplitude of spontaneous IPSCs by 29.9 ± 10.9% (*n*= 6; *P* < 0.05) without a change in frequency (TPEN: 6.6 ± 0.8 Hz, *n*= 6; *P* > 0.8) (Fig. 1*F*). However, analysis of large spontaneous events (>50 pA in four of six cells) (Fig. 1*G–I*) showed the frequency to be increased from 0.4 ± 0.1 Hz to 0.8 ± 0.2 Hz (*P* < 0.05) (Fig. 1*G* and *H*) and the amplitude by 20.3 ± 15.6 pA (*P* > 0.1) (Fig. 1*I*). These results suggest that local interneurones that mediate large spontaneous events may be predominantly modulated (Kraushaar & Jonas, 2000). In a separate set of control experiments, we obtained whole-cell recordings from CA3 pyramidal cells and recorded polysynaptic IPSCs evoked by a stimulus electrode positioned in stratum radiatum. Zinc chelation had no effect on evoked IPSCs (TPEN: 5.2 ± 5.1% amplitude decrease, *n*= 4; *P* > 0.3), which is consistent with the low zinc levels found in this hippocampal subdivision. Altogether, these results argue that endogenous zinc has a substantial influence on mono- and di-synaptic GABAergic transmission to granule cells.

### Blocking T-type Ca^2+^ channels occludes the effect of zinc chelation

**Figure 2:**
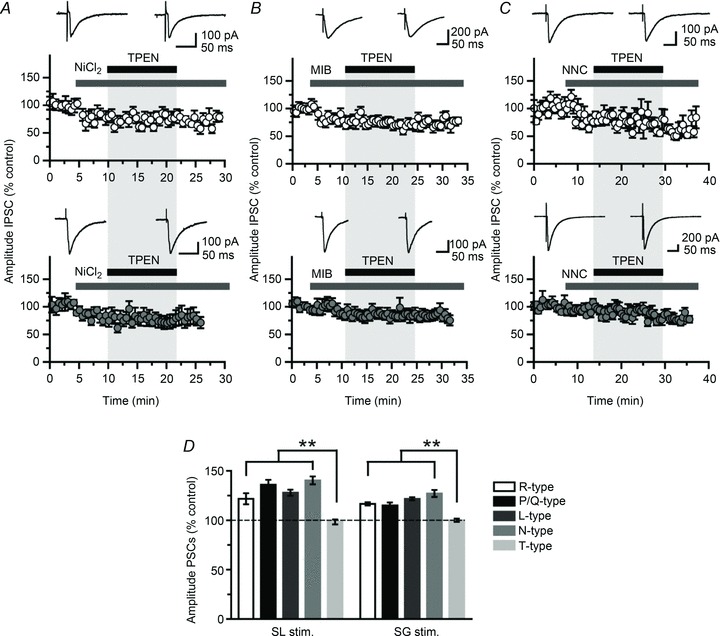
Blocking T-type Ca^2+^ channels occludes the effect of zinc chelation *A*, plots of IPSC amplitude against time showing that zinc chelation with TPEN (1 μm) has no effect when T-type Ca^2+^ channels are blocked by a low concentration of NiCl_2_ (10 μm; data from 11 neurones). *B*, *C*, similar experiment as in *A* but in the presence of the T-type Ca^2+^ channel antagonists mibefradil (10 μm) or NNC 55-0396 (10 μm). Again, chelation of zinc with TPEN (1 μm) has no effect. (Data are from nine and seven neurones, respectively.) Grey areas indicate that the effects of drug application are monitored simultaneously at both pathways. *D*, effects of zinc chelation against a background of Ca^2+^ channel blockers. (SNX-801 for R-type; 0.5 μm, *n*= 4; ω-agatoxin IVA for P/Q-type; 2 μm, *n*= 4; nifedipine for L-type; 20 μm, *n*= 3; ω-conotoxin GVIA for N-type; 2 μm, *n*= 3; NiCl_2_, mibefradil and NNC 55-0386 for T-type; 10 μm, *n*= 27). (**P< 0.01, ANOVA.)

Because the present results mainly show tonic effects, they do not shed light on any cell type or synapse harbouring the modulation. One possibility is that zinc binds relatively unselectively to Ca^2+^ channels (Busselberg *et al.* 1994; Brandt *et al.* 2005), thus depressing GABA release from presynaptic interneurones. In particular, hippocampal and neocortical interneurones express T-type Ca^2+^ channels (Goldberg *et al.* 2004; McKay *et al.* 2006), which are sensitive to zinc when expressed in heterologous systems (Traboulsie *et al.* 2007). We thus recorded from slices treated with Ca^2+^ channel blockers and repeated the chelator experiments (Fig. 2). We first analysed the effect of a low concentration of nickel (NiCl_2_, 10 μm) (Fig. 2*A*). Although NiCl_2_ has been reported to block T-type Ca^2+^ channels (Ca_v_3.n), it can also block R-type Ca^2+^ currents (Magee & Johnston, 1995). We found that the facilitation of evoked IPSCs was no longer observed when TPEN (1 μm) was applied in the background of NiCl_2_ (stratum lucidum stimulation: 1.6 ± 2.5% IPSC amplitude reduction, *n*= 11; stratum granulosum stimulation: 0.2 ± 1.6% change, *n*= 9; *P* > 0.4). We also tested mibefradil (10 μm), which is most selective for T-type Ca^2+^ channels (McDonough & Bean, 1998) but can also block R-type channels (Ca_v_2.n) at a low concentration and other voltage-gated channels at higher concentrations (Perez-Reyes, 2003). Again, we found that the facilitation of evoked IPSCs was absent when TPEN (1 μm) was applied on a background of mibefradil (stratum lucidum stimulation: 1.1 ± 4.9% IPSC amplitude reduction, *n*= 7; stratum granulosum stimulation: 4.7 ± 2.9% increase, *n*= 9; *P* > 0.4) (Fig. 2*B*). Finally, we applied the potent inhibitor of T-type Ca^2+^ channels containing Ca_v_3.1 subunits (NNC 55-0396, 10 μm) (Huang *et al.* 2004) and found similar results (stratum lucidum stimulation: 2.7 ± 7.7% IPSC amplitude increase; stratum granulosum stimulation: 8.3 ± 3.0% decrease, *n*= 7; *P* > 0.1) (Fig. 2*C*). Interestingly, T-type Ca^2+^ channel blockers reduced the amplitude of evoked IPSCs to approximately similar levels (NiCl_2_, stratum lucidum stimulation: 22.1 ± 5.0%*versus* stratum granulosum stimulation: 24.8 ± 5.5%, *n*= 7; mibefradil, stratum lucidum stimulation: 21.8 ± 4.4%*versus* stratum granulosum stimulation: 10.8 ± 4.1%, *n*= 7; NNC 55-0396, stratum lucidum stimulation: 22.8 ± 3.7%*versus* stratum granulosum stimulation: 6.8 ± 3.5%, *n*= 7; *P* > 0.1) (Fig. 2*A–C*). Recordings performed in slices treated with SNX-482 (0.5 μm, *n*= 4), a selective antagonist of α_1E_-containing R-type (Ca_v_2.3) channels, or the L-type Ca^2+^ channel antagonist nifedipine (20 μm, *n*= 3) did not differ from those obtained in control slices (ANOVA, *P* > 0.05). Furthermore, blocking P/Q-type Ca^2+^ channels with ω-agatoxin IVA (0.5 μm, *n*= 4) or N-type Ca^2+^ channels with ω-conotoxin GVIA (2 μm, *n*= 3) had no effect (ANOVA, *P* > 0.05) (Fig. 2*D*). These data argue that L-, P/Q- or N-type Ca^2+^ channels play a minor role and that zinc-mediated inhibition of T-type Ca^2+^ channels modulates evoked IPSCs in granule cells.

### Extracellular zinc chelation enhances dentate interneurone excitability

**Figure 3:**
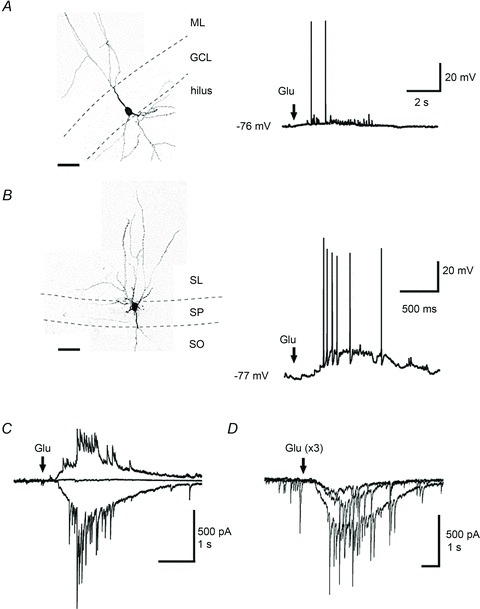
Focal glutamate application activates local interneurones *A*, current-clamp recording from a dentate interneurone stained with biocytin showing action potentials in response to focal glutamate application in stratum moleculare (arrow). *B*, a similar puff in stratum lucidum evokes action potentials in a different interneurone. GCL, granule cell layer; ML, molecular layer; SL, stratum lucidum; SP, stratum pyramidale; SO, stratum oriens. Dashed lines indicate layer boundaries. *C*, superimposed synaptic currents evoked by three glutamate puffs in stratum granulosum while recording from a granule cell held at −70 mV (inward), 0 mV (no current) or +40 mV (outward). *D*, superimposed traces showing three responses evoked by glutamate puffs of increased duration (30–50 ms). Calibration bars for confocal observations: 50 μm.

**Figure 4:**
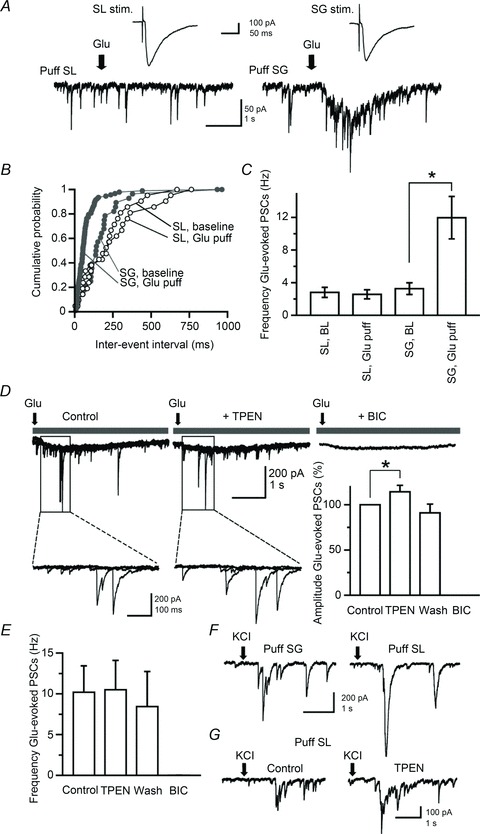
Glutamate puff in stratum granulosum elicits bursts of IPSCs in granule cells, the amplitude of which increases after chelation of zinc *A*, electrical stimulation in stratum lucidum or stratum granulosum evokes PSCs in a granule cell (top traces), whereas chemical activation of local interneurones with glutamate puffs elicits PSCs only if they are delivered in stratum granulosum (bottom traces). Arrows indicate the onset of the glutamate puff (30 p.s.i., 50 ms). *B*, cumulative distribution of PSC frequency in the same cell before (baseline) and after glutamate puffs in stratum lucidum and stratum granulosum. *C*, data pooled from six neurones showing the increase in PSC frequency after focal glutamate application in stratum granulosum. *D*, sample recordings obtained in one neurone (superimpositions of four consecutive trials) showing the facilitation of Glu-evoked PSCs after chelation of zinc with TPEN (1 μm). Glu-evoked PSCs are abolished by the addition of bicuculline (BIC; 10 μm). Selected portions of the traces are shown at higher magnification (bottom). The bar graph summarizes data obtained in six neurones (*P < 0.05, paired *t* test). *E*, the effect of TPEN in the same neurones is not accompanied by a change in glutamate-evoked PSC frequency. *F*, *G*, sample traces taken from two recorded neurones showing that focal KCl application (arrows; 50 p.s.i., 50 ms) evokes bursts of PSCs whether delivered in stratum granulosum or stratum lucidum. Focal KCl application in stratum lucidum elicits bursts of PSCs that are enhanced by superfusion of TPEN (1 μm).

**Figure 5:**
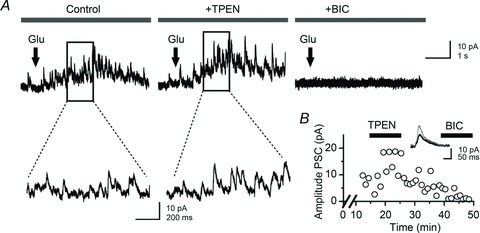
Blocking excitatory synaptic transmission in the recorded granule cell does not alter the facilitation of glutamate-evoked IPSCs *A*, sample recordings from a granule cell held at 0 mV and filled with a Cs-gluconate-based solution. Glutamate puffs in stratum granulosum (30 p.s.i., 50 ms; arrows) elicit bursts of IPSCs that are outward (left). Superfusion of TPEN (1 μm) increases the amplitude of glutamate-evoked IPSCs (middle) and bicuculline (10 μm) abolishes them (right.) Expanded portions of the traces are shown at higher magnification. *B*, plot of PSC amplitude against time (*x*-axis is cut) in one neurone showing a reversible enhancement after zinc chelation with TPEN (1 μm) and suppression by addition of bicuculline methiodide (10 μm). Averaged IPSCs (10 consecutive trials, superimposed) are shown before (black) and after (grey) superfusion of TPEN.

Where are the synapses affected by zinc chelators and by which presynaptic neurones are they formed? The facilitation of stratum lucidum evoked IPSCs is consistent with an effect at mossy fibre–CA3 interneurone synapses or, alternatively, synapses made onto dentate interneurones by recurrent mossy fibres and axon terminals from hilar mossy cells. Such interneurones also have excitatory synapses that show the peculiar metabotropic receptor agonist sensitivity of mossy fibres and extend their axons in stratum lucidum (Alle *et al.* 2001). To identify the location of presynaptic interneurones, we pressure-applied glutamate via a pipette positioned close to a site at which electrical stimulation evoked a di-synaptic IPSC in granule cells. d-APV was omitted from the perfusion solution in order to allow excitation of dendritic NMDA receptors (Weisskopf *et al.* 1993) in interneurones, whereas NBQX (20 μm) was included to minimize polysynaptic activity. We first performed control experiments in current-clamp to verify that pressure-applied glutamate in stratum lucidum (*n*= 3) or in the dentate molecular layer (*n*= 2) could recruit local interneurones (Fig. 3*A* and *B*). Further control experiments in granule cells held in voltage-clamp at −70 mV with a Cs-Cl containing pipette revealed IPSCs that reversed at 0 mV in response to glutamate puffs delivered >300 μm in the layer (Fig. 3*C* and *D*). These responses are likely to reflect the polysynaptic activation of GABA_A_ receptors in granule cells consecutive to firing in local interneurones. When applied in various locations near the stratum lucidum stimulus site, the glutamate puff failed to elicit IPSCs in granule cells, in contrast to electrical stimuli (Fig. 4*A–C*). However, in recordings from the same cells with the puff pipette repositioned in the dentate gyrus near the stratum granulosum stimulus electrode, glutamate puffs elicited bursts of IPSCs, the amplitude of which increased by 14.6 ± 7.1% following the superfusion of TPEN (1 μm, *n*= 6; *P* < 0.05) (Fig. 4*C* and *D*). TPEN did not affect the frequency of IPSCs evoked by such glutamate puffs (Δ_Fq_= 0.1 ± 1.4 Hz, *n*= 6; *P* > 0.05) (Fig. 4*E*). In a separate set of experiments (*n*= 4), glutamate was replaced by KCl (3 m) on the assumption that a local build-up in extracellular K^+^ would depolarize mossy fibres and thus mimic electrical stimulation. KCl puffs elicited bursts of IPSCs in granule cells irrespective of the site of application (Fig. 4*F*) and superfusion of TPEN (1 μm) increased their amplitude (Fig. 4*G*). A similar enhancement of glutamate-evoked responses by TPEN was obtained when holding granule cells near the reversal potential for glutamate receptors with a Cs-gluconate-based pipette solution (*V*_holding_= 0 mV, *E*_Cl_=−70.3 mV; *n*= 4) (Fig. 5). Altogether, these experiments show that dentate interneurones with terminals that release GABA onto granule cells can be recruited by focal glutamate application or action potentials in mossy fibre collaterals and that zinc modulates this process.

**Figure 6:**
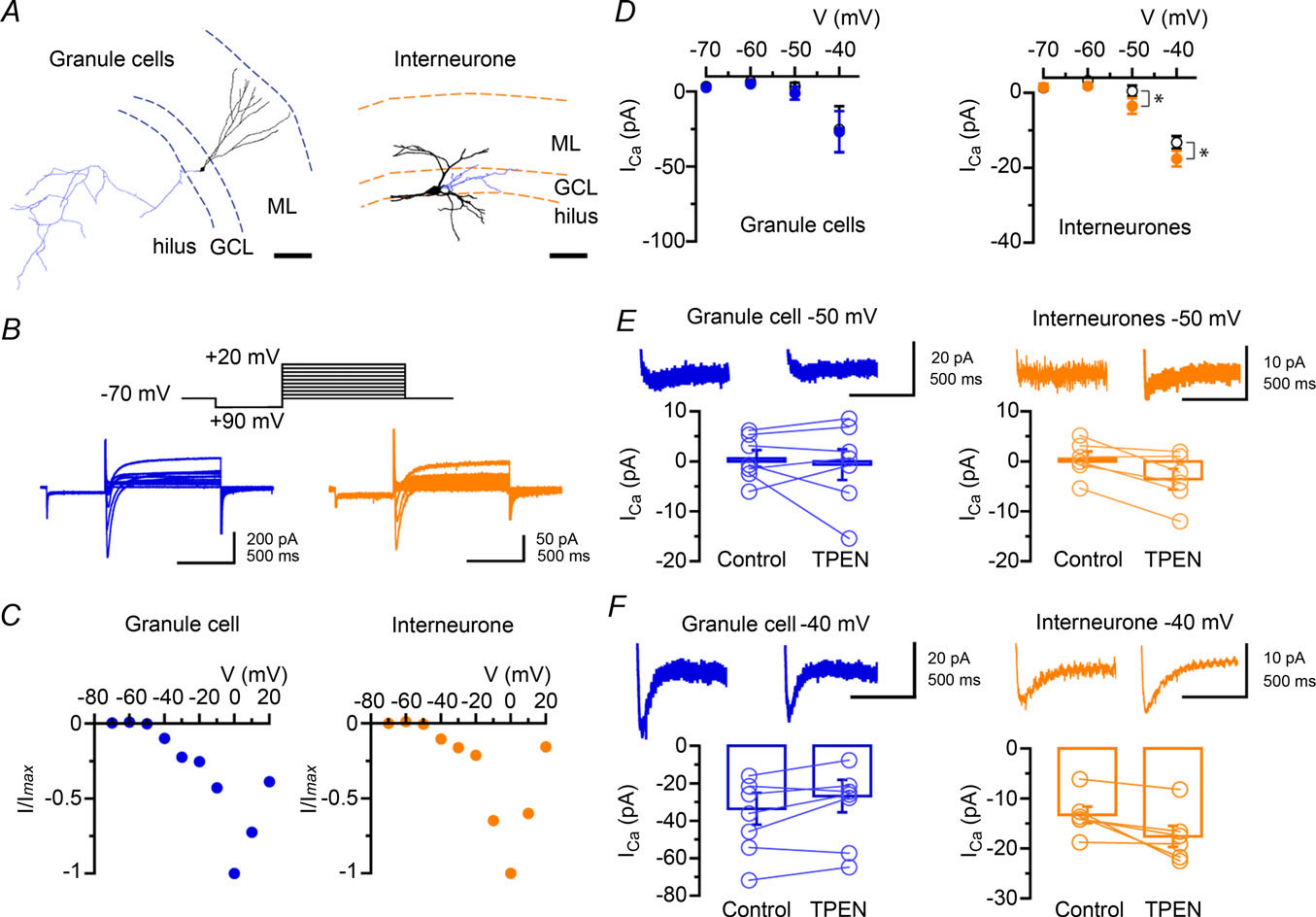
Zinc chelation facilitates T-type Ca^2+^ currents in fast-spiking interneurones *A*, digital reconstruction of a granule cell forming a network of mossy fibre collaterals within the hilus (left) and of a dentate interneurone (right), the cell body of which is located at the border between the granular layer and the hilar region. Axonal arbours are coloured in blue. The location of the cells is shown in relation to the different dentate layer boundaries. GCL, granule cell layer; ML, molecular layer. Scale bars: 100 μm. *B*, examples of *I*–*V* relations in a granule cell (left) and a dentate interneurone (right) showing the activation of a transient inward current evoked by depolarizing voltage steps. The voltage step protocol is represented above traces. *C*, normalized peak current amplitude plotted against the pipette potential for cells shown in *B*. *D*, zinc chelation with TPEN (1 μm) selectively enhances the amplitude of low-voltage activated T-type Ca^2+^ currents in fast-spiking interneurones (*n*= 7, right), but not granule cells (*n*= 8, left) (*P < 0.05, Student’s paired *t* test). *E*, *F*, summary bar graphs and data from individual neurones showing the effects of TPEN on the amplitude of Ca^2+^ currents at −50 mV (*E*) and −40 mV (*F*) in granule cells (*n*= 7, left) and interneurones (*n*= 6, right). Averages of three sweeps at each potential are shown for the control condition and TPEN (traces were truncated for clarity).

**Figure 7:**
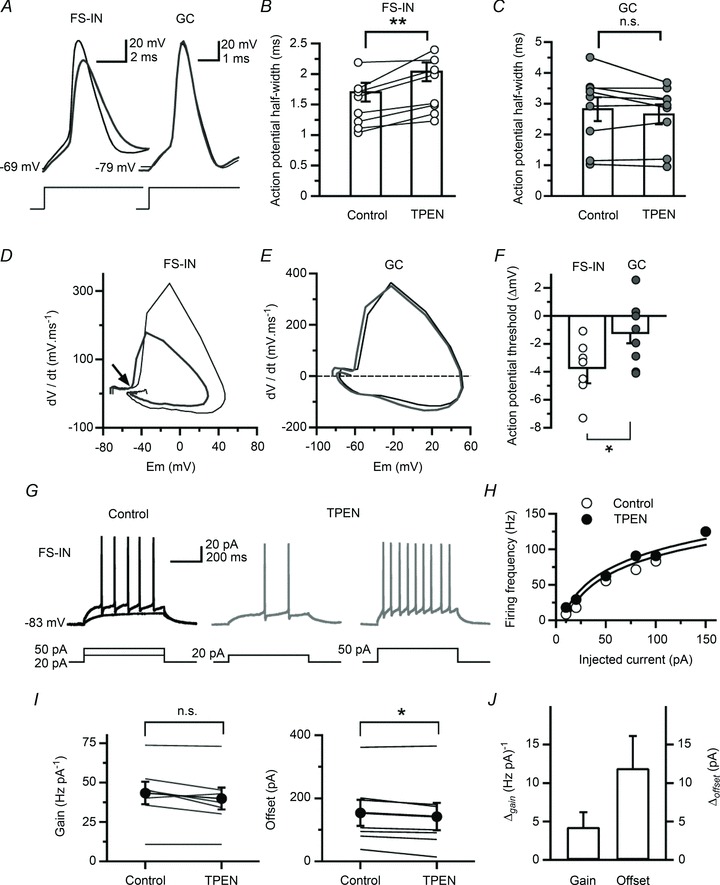
Zinc chelation modulates the action potential waveform and the offset of the I–O relation in fast-spiking interneurones but not granule cells *A*, superimposed action potential waveforms (averages of 20 sweeps) in a fast-spiking interneurone and a granule cell, in the control condition (thin black trace) and after chelation of zinc with TPEN (1 μm; thick grey trace). *B*, *C*, bar graphs summarize the broadening of action potentials in all fast-spiking interneurone recordings (*n*= 8) and no effect in granule cells (*n*= 9) (**P < 0.01, Student’s paired *t* test). *D*, *E*, time-derivative of the action potentials displayed in *A*, plotted against the membrane potential. The thin black trace is the phase plot in control condition and the thick grey trace is that after the addition of TPEN (1 μm). The arrow denotes the shift of the action potential threshold towards negative values. *F*, summary data showing the effect of TPEN (1 μm) on the action potential threshold in fast-spiking interneurone recordings (*n*= 8) and the absence of effect in granule cells (*n*= 9) (*P < 0.05, Mann–Whitney *U* test.) *G*, examples traces obtained in a fast-spiking interneurone showing voltage deflections in response to depolarizing current steps that are sub- and supra-threshold to action potential initiation (control). A rheobase current of +20 pA elicited two action potentials, whereas injection of +50 pA triggered nine action potentials in the presence of TPEN (1 μm; grey traces). *H*, example of I–O relation obtained in a fast-spiking interneurone showing a shift in offset but no change in gain. *I*, effects of TPEN (1 μm) on the gain and offset of the I–O relation in fast-spiking interneurones (*n*= 7) (*P < 0.05, Student’s paired *t* test; n.s., non-significant.) *J*, as in *I* but expressed as a relative change.

Dentate interneurones provide a strong inhibitory input to granule cells and receive zinc-containing boutons (Ribak *et al.* 1990; Ribak & Peterson, 1991). We recorded from interneurones located at the border between the granule cell layer and the polymorphic layer of the hilus, systematically labelled them with biocytin and analysed the zinc sensitivity of their pharmacologically isolated low-voltage activated Ca^2+^ currents. Similar experiments were performed in granule cells for comparison (Fig. 6*A*). Confocal analysis of Z-stack projections from recorded interneurones revealed a large soma size (35–45 μm), thick apical and basal dendritic trunks through the granular region and extending towards the stratum moleculare and the hilus, and axonal projections that ramified in the granular layer, with the exception of one cell, which had axon ramifications in stratum moleculare. Granule cells and dentate interneurones were held at *V*_holding_=−70 mV and a 500 ms pre-pulse followed by a 1 s step from −90 mV to +20 mV with 10 mV increments was applied, in the presence of a cocktail of channel blockers and ionotropic receptor antagonists (see Methods). This protocol activated transient inward currents, the amplitude of which peaked and declined when the pipette potential was >0 mV (Fig. 6*B* and *C*). Stepping the pipette voltage to −40 mV elicited transient Ca^2+^ currents in granule cells (−36.4 ± 7.8 pA, *n*= 8) and interneurones (−13.3 ± 3.9 pA, *n*= 7) (Fig. 6*D* and *F*). Zinc chelation increased peak currents in interneurones (control: −13.3 ± 1.6 pA *versus* TPEN: −17.6 ± 2.1 pA, *n*= 6; *P* < 0.04), but had no effect on those recorded in granule cells (control: −33.6 ± 8.5 pA *versus* TPEN: −26.8 ± 8.7 pA, *n*= 7; *P* > 0.05). Application of mibefradil (10 μm) reduced their amplitude to −7.0 ± 11.2 pA (granule cells, *n*= 5; *P* < 0.04) and −3.1 ± 4.2 pA (interneurones, *n*= 4; *P* < 0.05) confirming Ca^2+^ influx through T-type Ca^2+^ channels (data not shown). To relate these changes to effects on interneurone excitability, we switched to current-clamp mode and recorded voltage responses elicited by hyperpolarizing and depolarizing current steps (data summarized in Table 1). TPEN (1 μm) did not alter the membrane potential *V*_m_, the input resistance *R*_in_, the membrane time-constant *τ*_m_ or the maximum firing rate in either granule cells or fast-spiking interneurones, and neither did it change spike peak amplitude in fast-spiking interneurones (control: 110.9 ± 5.3 mV; TPEN: 108.1 ± 4.8 mV, *n*= 8; *P* > 0.3) or granule cells (control: 105.2 ± 3.9 mV; TPEN: 101.6 ± 5.3 mV, *n*= 9; *P* > 0.08). However, TPEN selectively prolonged spike width in fast-spiking interneurones (control: 1.5 ± 0.1 ms; TPEN: 1.8 ± 0.2 ms, *n*= 8; *P* < 0.004) (Fig. 7*A*) without affecting that measured in granule cells (control: 2.8 ± 0.4 ms; TPEN: 2.7 ± 0.3 ms, *n*= 9; *P* > 0.2) (Fig. 7*B*). The effect on spike duration in fast-spiking interneurones was accompanied by a reduction in spike threshold from −40.2 ± 3.1 mV to −43.8 ± 3.3 mV (*n*= 8; *P* < 0.001) (Fig. 7*D* and *F*). Again, this was not the case in recordings from granule cells (control: −46.1 ± 2.4 mV; TPEN: −47.3 ± 2.2 mV, *n*= 9; *P* > 0.1) (Fig. 7*E* and *F*). Comparisons of spike width and threshold in granule cells and fast-spiking interneurones yielded significant differences (*P* < 0.04, Mann–Whitney *U* test). Furthermore, TPEN selectively lowered the rheobase current (i.e. the injected current required to reach the action potential threshold) in six of seven fast-spiking interneurones. As Fig. 7*G* shows, a depolarizing current step of +50 pA was required to elicit five action potentials in a fast-spiking interneurone, whereas in the presence of TPEN (1 μm) this current was +20 pA, triggering two action potentials. Injection of the rheobase current elicited more action potentials against a background of TPEN than in the control condition (spike count, control: 3.9 ± 1.6 *versus* TPEN: 6.7 ± 1.6, *n*= 8; *P* < 0.05) suggesting enhanced excitability. Fitting the I–O relation with a logarithmic function (Mitchell & Silver, 2003; Kerr & Capogna, 2007) unveiled a reduction in neuronal offset in fast-spiking interneurones (control: 154.1 ± 41.3 pA; TPEN: 142.2 ± 43.3 pA; *n*= 7; *P* < 0.03) without change in neuronal gain (control: 43.4 ± 7.1 Hz*pA^−1^*versus* TPEN: 39.9 ± 6.9 Hz*pA^−1^; *n*= 7; *P* > 0.05) (Fig. 7*H*–*J*). In contrast, the number of spikes at rheobase current remained unchanged in granule cells (spike count, control: 2.9 ± 0.9 *versus* TPEN: 2.6 ± 1.2; *n*= 6; *P* > 0.05). In summary, these data show that removal of the chelatable zinc fraction enhanced T-type Ca^2+^ channel activity and increased cellular excitability in fast-spiking interneurones without affecting the properties of granule cells.

**Table 1:** Lack of effect of zinc chelation on the electrical properties of granule cells (*n*= 9) and fast-spiking interneurones (FS-INs, *n*= 8)

	Granule cells		FS-INs	
	Control	TPEN	Control	TPEN
*V*_m_ (mV)	−88.8 ± 3.7	−91.3 ± 3.0	−77.1 ± 2.9	−79.2 ± 3.0
*R*_input_ (MΩ)	326 ± 47	335 ± 59	396 ± 69	445 ± 79
Sag ratio	0.98 ± 0.01	0.971 ± 0.02	0.94 ± 0.06	0.95 ± 0.09
Amplitude AP (mV)	105.2 ± 3.9	101.6 ± 5.3	110.9 ± 5.3	108.1 ± 4.8
Firing frequency, max (s^−1^)	100.1 ± 11.2	101.4 ± 9.1	131.0 ± 9.0	130.0 ± 12.0

### Endogenous zinc broadens the time window for integration of perforant path inputs

**Figure 8:**
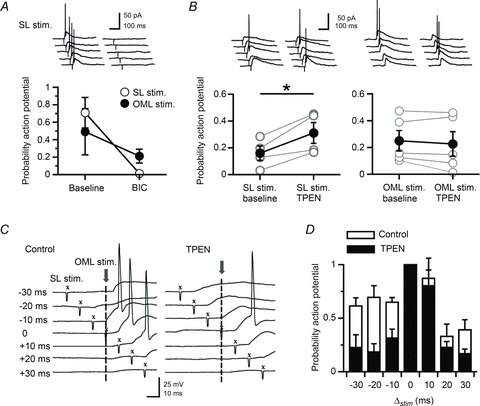
Zinc chelation narrows the time window for integration of perforant path inputs *A*, blocking GABA_A_ receptors with bicuculline (10 μm) blocks action potentials evoked by stimuli delivered in stratum lucidum. *B*, conversely, chelation of zinc with TPEN (1 μm) increases this probability. (*P < 0.05, Student’s paired *t* test.) TPEN does not affect the probability for evoking action potentials elicited by stimulation of the outer molecular layer (*P* > 0.05, Student’s paired *t* test). (Data presented from five neurones.) *C*, representative voltage traces taken from one neurone at different inter-stimulus intervals (−30 ms to +30 ms) in the control condition and after chelation of zinc with TPEN (1 μm). Vertical dashed lines indicate the time reference (*t*= 0) when electrical stimuli were delivered in the outer molecular layer. X, stratum lucidum stimulation. *D*, summary histogram showing the narrowing of the window for integration of perforant path inputs in the presence of TPEN (data from five neurones). The probability for evoking action potentials by stimulating the perforant path is reduced for inter-stimulus intervals >10 ms (*P* < 0.05, ANOVA).

Synaptic inhibition in dentate granule cells mainly operates by the shunt of concurrently activated excitatory inputs caused by the reduction in membrane resistivity and dendritic space constant associated with the GABA_A_ receptor-mediated conductance (Rall, 1964; Barrett & Crill, 1974). Furthermore, the reversal potential for GABA_A_ receptor activation is ∼16 mV more depolarized than the resting membrane potential (*E*_resting_=−82.5 ± 2.7 mV) (see Misgeld *et al.* 1986; Soltesz & Mody, 1994), which implies that GABAergic events remain largely depolarizing. Because mossy fibre collaterals can rapidly recruit fast-spiking interneurones, ensuring a precise temporal integration of the excitatory input (Geiger *et al.* 1997; Calixto *et al.* 2008), we tested the hypothesis that zinc-mediated modulation of interneurone activity regulates synaptic integration in granule cells and ultimately spike routing to CA3. We positioned a tungsten electrode in stratum lucidum to stimulate mossy fibres and another in the outer molecular layer to stimulate the lateral perforant path. When recording from granule cells in current-clamp mode, electrical stimulation delivered in stratum lucidum elicited a depolarizing potential able to initiate action potentials. Superfusion of bicuculline (10 μm, *n*= 3) abolished this synaptic response and firing (Fig. 8*A*) in a manner similar to the effect of blocking AMPA/kainate receptors with NBQX (20 μm, *n*= 3; data not shown) and in good agreement with the data obtained in the voltage-clamp configuration (Fig. 1*C* and *E*). These observations were consistent with di-synaptic GABAergic inputs to granule cells that were conveyed by mossy fibre collateral–interneurone synapses, the depolarizing nature of which could trigger action potentials. They also revealed some degree of feed-forward activation of inhibitory neurones by the outer molecular layer stimulus. When chelating zinc with TPEN (1 μm) the probability for evoking action potentials in granule cells increased from 0.16 ± 0.05 to 0.31 ± 0.08 (*n*= 4; *P* < 0.04) without affecting action potentials generated by the activation of lateral perforant path synapses (Fig. 8*B*). We adjusted the stimulus intensities so that simultaneous activation of the two pathways resulted in an approximately 50% chance of the neurone spiking (Fig. 8*C*). We then measured the spike probability while systematically varying the inter-stimulus interval. (Negative inter-stimulus intervals indicate that stratum lucidum stimuli preceded those delivered in the outer molecular layer.) As reported in CA1 pyramidal cells (Pouille & Scanziani, 2004; Pavlov *et al.* 2011), spike probability decreased as the interval increased. We then superfused TPEN (1 μm), which resulted in a significant narrowing of the time window for the integration of perforant path inputs at inter-stimulus intervals of >10 ms (*n*= 5; ANOVA, *P* < 0.05) (Fig. 8*D*).

**Figure 9:**
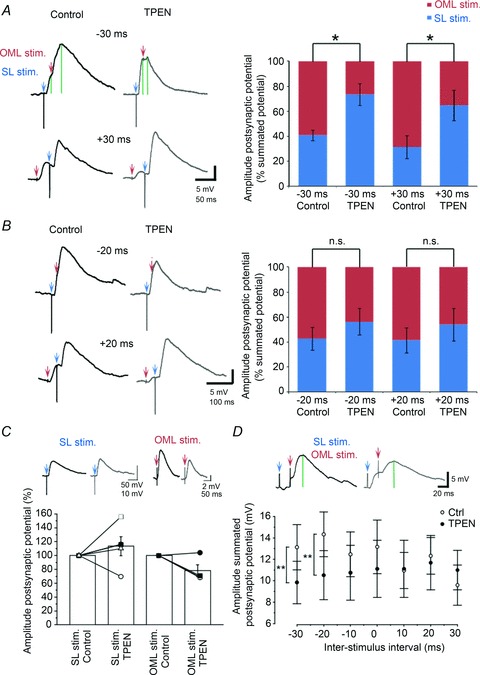
Zinc chelation reduces the contribution of glutamatergic perforant path inputs to summated subthreshold potentials *A*, *B*, summated postsynaptic potentials (averages of five consecutive sweeps) evoked by stratum lucidum stimulation (SL stim, blue arrow) and outer molecular layer stimulation (OML stim, red arrow) at two different inter-stimulus intervals (−30/+30 ms and −20/+20 ms), in control condition (black traces) and in the presence of TPEN (grey traces). Green vertical lines on top traces indicate the peak of the first and summated responses. Bar graphs show amplitudes of individual responses at both pathways expressed as percentages of the summated responses, in the control condition and following chelation of zinc with TPEN (*P < 0.05, ANOVA). *C*, summary bar graph and data from individual experiments showing the effect of zinc chelation on individual responses. *D*, amplitude of the summated potential at different inter-stimulus intervals, in the control condition (white symbols) and after superfusion of TPEN (black symbols). **P < 0.01, Student’s *t* test). Data presented are from five neurones.

A possible explanation for this finding may refer to the increased shunting of perforant path-mediated EPSPs by the di-synaptic GABA_A_ receptor-mediated conductance activated by recurrent mossy fibre collaterals forming synapses onto interneurones. To examine whether a shunting mechanism might explain the narrowing of the window for the integration of perforant path inputs, we analysed the contribution of each pathway to the overall subthreshold summated postsynaptic potential, before and after zinc chelation. The contribution of lateral perforant path inputs to the summated postsynaptic potential was significantly smaller after superfusion of TPEN (−30 ms, control: 59.1 ± 4.1%*versus* TPEN: 26.6 ± 8.7%; +30 ms, control: 68.5 ± 8.9%*versus* TPEN: 35.1 ± 12.3%; −20 ms, control: 57.2 ± 9.1%*versus* TPEN: 43.7 ± 10.4%; +20 ms, control: 58.5 ± 9.9%*versus* TPEN: 45.9 ± 13.0%), whereas that of postsynaptic potentials evoked by stratum lucidum stimulation became larger (−30 ms, control: 40.9 ± 4.1%*versus* TPEN: 73.4 ± 8.7%; +30 ms, control: 31.4 ± 8.9%*versus* TPEN: 64.9 ± 12.3%; −20 ms, control: 42.8 ± 9.1%*versus* TPEN: 56.3 ± 10.4%; +20 ms, control: 41.5 ± 9.9%*versus* TPEN: 54.1 ± 13.0%), implying a shift in the excitation–inhibition balance (Fig. 9*A*). This change was greater for large intervals of ±30 ms (*n*= 5; ANOVA, *P* < 0.05) and could still be observed at intervals of ±20 ms, albeit non-significantly (Fig. 9*B*). The amplitude of individual responses was increased by 13.5 ± 10.7% (stratum lucidum stimulation) and decreased by 21.9 ± 8.7% (outer molecular layer stimulation) (*n*= 5; *P* > 0.05) (Fig. 9*C*). Finally, for intervals in which stratum lucidum stimulation preceded that delivered in the outer molecular layer, the amplitude of the summated response was significantly smaller in the presence of TPEN (e.g. −20 ms, control: 14.3 ± 2.1 mV *versus* TPEN: 10.5 ± 2.3 Mv; *P* < 0.05) (Fig. 9*D*). This observation pointed towards a shunting effect of the di-synaptic GABAergic conductance. Although these experiments do not directly link the changes in granule cell excitability to those happening in presynaptic interneurones, they underline the fact that zinc facilitates granule cell excitability depending on the timing of the combined activation of a glutamatergic monosynaptic input and a di-synaptic GABAergic input, respectively. Thus, endogenous zinc regulates information transfer to local targets in the dentate gyrus with significant consequences on spike routing from granule cells to CA3.

## Discussion

We found that endogenous zinc depresses mono- and di-synaptic GABAergic signalling in granule cells by acting on T-type Ca^2+^ channels. We also found that zinc chelation selectively modulated action potential threshold properties in fast-spiking interneurones and that it narrowed the time window for integration of glutamatergic perforant path inputs in granule cells. It is highly unlikely that our findings of chelation-mediated modulation of dentate granule cell excitability can be explained by an effect on GABA_A_ receptors in somata and dendrites of granule cells themselves. Firstly, their somatic holding current was not affected by zinc chelation, which is consistent with the relatively low zinc sensitivity of GABA_A_ receptors in this cell type at this postnatal age (Buhl *et al.* 1996; Kapur *et al.* 1999). Secondly, the facilitation of evoked IPSCs consecutive to zinc chelation was of roughly the same order of magnitude whether a high- or low-chloride concentration was used in the pipette solution. Thirdly, zinc-mediated modulation of evoked GABAergic signalling in the dentate gyrus was absent if slices were treated with a T-type Ca^2+^ channel antagonist. Thus, zinc inhibits GABAergic signalling via direct binding to GABA_A_ receptors at a monosynaptic input (Ruiz *et al.* 2004) or by regulating the excitability of presynaptic interneurones at a di-synaptic pathway, as shown here. These findings imply that feed-forward and feedback inhibition in the dentate gyrus is tightly regulated by zinc. However, with the current experiment design, we cannot rule out the possibility that stratum lucidum stimulation recruited a proportion of back-projecting interneurones providing long-range cross-regional inhibition from CA1 to hilar regions (Sik *et al.* 1994; Szabadics & Soltesz, 2009). Lastly, the ultimate demonstration that the observed effects were directly linked to the vesicular release of zinc at mossy fibre–interneurone synapses would require the use of ZnT3^−/−^ mice, which lack the fraction of chelatable zinc from synaptic vesicles. Furthermore, we cannot exclude the possibility that non-vesicular release of zinc might contribute to the observed effects.

Our results showing that the T-type Ca^2+^ channel antagonists mibefradil and NNC 55-0396 reduced evoked GABAergic synaptic transmission and occluded the enhancing effect of zinc chelation suggests that zinc inhibits low-voltage activated Ca^2+^ channels in presynaptic dentate interneurones. In keeping with this, interneurones positioned in the inner molecular and granular layers show immunoreactivity for all Ca_v_3 isoforms from the T-type Ca^2+^ channel family (McKay *et al.* 2006; Vinet & Sik, 2006), let alone the dense localization of T-type Ca^2+^ channel gene transcripts in this area (Talley *et al.* 1999) and the rich innervation of parvalbumin-immunoreactive interneurones by Timm-stained mossy fibre collaterals (Blasco-Ibanez *et al.* 2000; Seress & Gallyas, 2000). Although T-type Ca^2+^ channels are generally not involved in evoked neurotransmitter release, they can initiate slow release from non-axonal sites (Cueni *et al.* 2009) and recent evidence in dorsal horn neurones (Jacus *et al.* 2012) and layer III enthorinal cortex (Huang *et al.* 2011) has shown that they can potently inhibit glutamate release. Ca_v_3.1-containing T-type Ca^2+^ channels can also modulate the release of quanta from GABAergic synapses formed by parvalbumin-expressing and perisomatic-targeting interneurones onto CA1 pyramidal cells (Tang *et al.* 2011). However, the precise molecular mechanisms responsible for the role of presynaptic T-type Ca_v_3.2 channels and their significance in evoked synaptic transmission would be best evaluated in interneurone–granule cell pairs in slices from transgenic mice that lack these channels.

Analysing spike shape and threshold as well as I–O relations revealed specific effects of zinc chelation in interneurones, but not in granule cells. The positive shift of neuronal offset in fast-spiking interneurones independently of gain was consistent with the removal of a fixed tonic conductance, the effect of which in the baseline condition dampened neuronal firing over the entire time domain. Whether zinc-mediated modulation of T-type Ca^2+^ channel activity caused the offset of the I–O relation in fast-spiking interneurones remains to be elucidated.

Our finding that TPEN narrowed the time window for the integration of excitatory synaptic inputs driven by lateral perforant path synapses has important ramifications for signal integration in the dentate gyrus. We showed that it was possible to position the stimulus electrode in stratum lucidum such that recurrent activation of mossy fibres would depolarize granule cells via di-synaptic GABAergic actions, as shown for monosynaptic connections (Chiang *et al.* 2012; Sauer *et al.* 2012). Activation of recurrent mossy fibre synapses and perforant path inputs evoked depolarizing postsynaptic potentials that summated, and yielded a significant increase in spiking probability in granule cells. When zinc was chelated with TPEN, a significant decrease in the contribution of perforant path inputs to the summated postsynaptic potential in favour of inputs activated by recurrent mossy fibres at stimulus intervals of >20 ms was observed. Taken together with the pro-excitatory effect of zinc chelation on granule cell firing (Fig. 6*A*), these results unravel a powerful homeostatic mechanism that tunes information transfer to dentate and CA3 microcircuits. Further, the modulation of dentate gyrus excitability by endogenous zinc has profound implications for developmental and pathological processes, in particular epilepsy, which is associated with an increase in the sensitivity of GABA_A_ receptors to inhibition by zinc, extensive mossy fibre sprouting and loss of interneurone populations.

## Abbreviations

DABCO: 1,4-diazabicyclo-[2,2,2]-octane
*E*_Cl_: reversal potential for chloride
*I*_holding_: holding current
*I*_max_: maximum intensity current
QX314 Br: *N*-ethyllidocaine bromide
PSC: postsynaptic current
*R*_input_: input resistance
*V*_holding_: holding potential
*V*_m_: membrane potential
ZnT3: zinc transporter type 3

## References

[b1] Acsady L, Kamondi A, Sik A, Freund T, Buzsaki G (1998). GABAergic cells are the major postsynaptic targets of mossy fibers in the rat hippocampus. J Neurosci.

[b2] Aizenman E, Stout AK, Hartnett KA, Dineley KE, McLaughlin B, Reynolds IJ (2000). Induction of neuronal apoptosis by thiol oxidation: putative role of intracellular zinc release. J Neurochem.

[b3] Alle H, Jonas P, Geiger JR (2001). PTP and LTP at a hippocampal mossy fibre–interneuron synapse. Proc Natl Acad Sci U S A.

[b4] Barberis A, Cherubini E, Mozrzymas JW (2000). Zinc inhibits miniature GABAergic currents by allosteric modulation of GABA_A_ receptor gating. J Neurosci.

[b5] Barrett JN, Crill WE (1974). Influence of dendritic location and membrane properties on the effectiveness of synapses on cat motoneurones. J Physiol.

[b6] Bartos M, Alle H, Vida I (2011). Role of microcircuit structure and input integration in hippocampal interneuron recruitment and plasticity. Neuropharmacology.

[b7] Bartos M, Vida I, Jonas P (2007). Synaptic mechanisms of synchronized gamma oscillations in inhibitory interneuron networks. Nat Rev Neurosci.

[b8] Bean BP (2007). The action potential in mammalian central neurons. Nat Rev Neurosci.

[b9] Berger T, Schwarz C, Kraushaar U, Monyer H (1998). Dentate gyrus basket cell GABA_A_ receptors are blocked by Zn^2+^ via changes of their desensitization kinetics: an *in situ* patch-clamp and single-cell PCR study. J Neurosci.

[b10] Blasco-Ibanez JM, Martinez-Guijarro FJ, Freund TF (2000). Recurrent mossy fibers preferentially innervate parvalbumin-immunoreactive interneurons in the granule cell layer of the rat dentate gyrus. Neuroreport.

[b11] Brandt A, Khimich D, Moser T (2005). Few Ca_v_1.3 channels regulate the exocytosis of a synaptic vesicle at the hair cell ribbon synapse. J Neurosci.

[b12] Buhl EH, Otis TS, Mody I (1996). Zinc-induced collapse of augmented inhibition by GABA in a temporal lobe epilepsy model. Science.

[b13] Busselberg D, Platt B, Michael D, Carpenter DO, Haas HL (1994). Mammalian voltage-activated Ca^2+^ channel currents are blocked by Pb^2+^, Zn^2+^, and Al^3+^. J Neurophysiol.

[b14] Buzsaki G (1984). Feed-forward inhibition in the hippocampal formation. Prog Neurobiol.

[b15] Calixto E, Galvan EJ, Card JP, Barrionuevo G (2008). Coincidence detection of convergent perforant path and mossy fibre inputs by CA3 interneurons. J Physiol.

[b16] Chiang PH, Wu PY, Kuo TW, Liu YC, Chan CF, Chien TC, Cheng JK, Huang YY, Chiu CD, Lien CC (2012). GABA is depolarizing in hippocampal dentate granule cells of the adolescent and adult rats. J Neurosci.

[b17] Cueni L, Canepari M, Adelman JP, Luthi A (2009). Ca^2+^ signalling by T-type Ca^2+^ channels in neurons. Pflugers Arch.

[b18] Doherty JJ, Alagarsamy S, Bough KJ, Conn PJ, Dingledine R, Mott DD (2004). Metabotropic glutamate receptors modulate feedback inhibition in a developmentally regulated manner in rat dentate gyrus. J Physiol.

[b19] Duce JA, Tsatsanis A, Cater MA, James SA, Robb E, Wikhe K, Leong SL, Perez K, Johanssen T, Greenough MA, Cho HH, Galatis D, Moir RD, Masters CL, McLean C, Tanzi RE, Cappai R, Barnham KJ, Ciccotosto GD, Rogers JT, Bush AI (2010). Iron-export ferroxidase activity of beta-amyloid precursor protein is inhibited by zinc in Alzheimer’s disease. Cell.

[b20] Ewell LA, Jones MV (2010). Frequency-tuned distribution of inhibition in the dentate gyrus. J Neurosci.

[b21] Freund TF, Buzsaki G (1996). Interneurons of the hippocampus. Hippocampus.

[b22] Geiger JR, Lubke J, Roth A, Frotscher M, Jonas P (1997). Submillisecond AMPA receptor-mediated signalling at a principal neuron–interneuron synapse. Neuron.

[b23] Gielen M, Siegler Retchless B, Mony L, Johnson JW, Paoletti P (2009). Mechanism of differential control of NMDA receptor activity by NR2 subunits. Nature.

[b24] Goldberg JH, Lacefield CO, Yuste R (2004). Global dendritic Ca^2+^ spikes in mouse layer 5 low threshold spiking interneurones: implications for control of pyramidal cell bursting. J Physiol.

[b25] Gu Y, Barry J, Gu C (2013). Kv3 channel assembly, trafficking and activity are regulated by zinc through different binding sites. J Physiol.

[b26] Hershfinkel M, Kandler K, Knoch ME, Dagan-Rabin M, Aras MA, Abramovitch-Dahan C, Sekler I, Aizenman E (2009). Intracellular zinc inhibits KCC2 transporter activity. Nat Neurosci.

[b27] Hosie AM, Dunne EL, Harvey RJ, Smart TG (2003). Zinc-mediated inhibition of GABA_A_ receptors: discrete binding sites underlie subtype specificity. Nat Neurosci.

[b28] Huang L, Keyser BM, Tagmose TM, Hansen JB, Taylor JT, Zhuang H, Zhang M, Ragsdale DS, Li M (2004). NNC 55-0396 [(1*S*,2*S*)-2-(2-(*N*-[(3-benzimidazol-2-yl)propyl]-*N*-methylamino)ethyl)-6-fluoro-1,2, 3,4-tetrahydro-1-isopropyl-2-naphtyl cyclopropanecarboxylate dihydrochloride]: a new selective inhibitor of T-type Ca^2+^ channels. J Pharm Exp Ther.

[b29] Huang YZ, Pan E, Xiong ZQ, McNamara JO (2008). Zinc-mediated transactivation of TrkB potentiates the hippocampal mossy fibre–CA3 pyramid synapse. Neuron.

[b30] Huang Z, Lujan R, Kadurin I, Uebele VN, Renger JJ, Dolphin AC, Shah MM (2011). Presynaptic HCN1 channels regulate Ca_v_3.2 activity and neurotransmission at select cortical synapses. Nat Neurosci.

[b31] Imbrici P, D’Adamo MC, Cusimano A, Pessia M (2007). Episodic ataxia type 1 mutation F184C alters Zn^2+^-induced modulation of the human K^+^ channel K_V_1.4–K_V_1.1/K_V_beta1.1. Am J Physiol Cell Physiol.

[b32] Jacus MO, Uebele VN, Renger JJ, Todorovic SM (2012). Presynaptic Ca_v_3.2 channels regulate excitatory neurotransmission in nociceptive dorsal horn neurons. J Neurosci.

[b33] Kapur J, Haas KF, Macdonald RL (1999). Physiological properties of GABA_A_ receptors from acutely dissociated rat dentate granule cells. J Neurophysiol.

[b34] Kerr AM, Capogna M (2007). Unitary IPSPs enhance hilar mossy cell gain in the rat hippocampus. J Physiol.

[b35] Kodirov SA, Takizawa S, Joseph J, Kandel ER, Shumyatsky GP, Bolshakov VY (2006). Synaptically released zinc gates long-term potentiation in fear conditioning pathways. Proc Natl Acad Sci U S A.

[b36] Koh DS, Geiger JR, Jonas P, Sakmann B (1995). Ca^2+^-permeable AMPA and NMDA receptor channels in basket cells of rat hippocampal dentate gyrus. J Physiol.

[b37] Kraushaar U, Jonas P (2000). Efficacy and stability of quantal GABA release at a hippocampal interneuron–principal neuron synapse. J Neurosci.

[b38] Magee JC, Johnston D (1995). Synaptic activation of voltage-gated channels in the dendrites of hippocampal pyramidal neurons. Science.

[b39] Mayer ML, Vyklicky L (1989). The action of zinc on synaptic transmission and neuronal excitability in cultures of mouse hippocampus. J Physiol.

[b40] McDonough SI, Bean BP (1998). Mibefradil inhibition of T-type Ca^2+^ channels in cerebellar Purkinje neurons. Mol Pharmacol.

[b41] McKay BE, McRory JE, Molineux ML, Hamid J, Snutch TP, Zamponi GW, Turner RW (2006). Ca_v_3 T-type Ca^2+^ channel isoforms differentially distribute to somatic and dendritic compartments in rat central neurons. Eur J Neurosci.

[b42] Misgeld U, Deisz RA, Dodt HU, Lux HD (1986). The role of chloride transport in postsynaptic inhibition of hippocampal neurons. Science.

[b43] Mitchell SJ, Silver RA (2003). Shunting inhibition modulates neuronal gain during synaptic excitation. Neuron.

[b44] Molnar P, Nadler JV (2001a). Synaptically-released zinc inhibits *N*-methyl-d-aspartate receptor activation at recurrent mossy fibre synapses. Brain Res.

[b45] Molnar P, Nadler JV (2001b). Lack of effect of mossy fibre-released zinc on granule cell GABA_A_ receptors in the pilocarpine model of epilepsy. J Neurophysiol.

[b46] Mott DD, Benveniste M, Dingledine RJ (2008). pH-dependent inhibition of kainate receptors by zinc. J Neurosci.

[b47] Myatt DR, Hadlington T, Ascoli GA, Nasuto SJ (2012). Neuromantic – from semimanual to semi-automatic reconstruction of neuron morphology. Front Neuroinform.

[b48] Nozaki C, Vergnano AM, Filliol D, Ouagazzal AM, Le Goff A, Carvalho S, Reiss D, Gaveriaux-Ruff C, Neyton J, Paoletti P, Kieffer BL (2011). Zinc alleviates pain through high-affinity binding to the NMDA receptor NR2A subunit. Nat Neurosci.

[b49] Paoletti P, Perin-Dureau F, Fayyazuddin A, Le Goff A, Callebaut I, Neyton J (2000). Molecular organization of a zinc binding n-terminal modulatory domain in a NMDA receptor subunit. Neuron.

[b50] Paoletti P, Vergnano AM, Barbour B, Casado M (2009). Zinc at glutamatergic synapses. Neuroscience.

[b51] Pavlov I, Scimemi A, Savtchenko L, Kullmann DM, Walker MC (2011). I(h)-mediated depolarization enhances the temporal precision of neuronal integration. Nat Commun.

[b52] Penttonen M, Kamondi A, Sik A, Acsady L, Buzsaki G (1997). Feed-forward and feed-back activation of the dentate gyrus *in vivo* during dentate spikes and sharp wave bursts. Hippocampus.

[b53] Perez-Reyes E (2003). Molecular physiology of low-voltage-activated T-type Ca^2+^ channels. Physiol Rev.

[b54] Pouille F, Scanziani M (2004). Routing of spike series by dynamic circuits in the hippocampus. Nature.

[b55] Rall W, Reiss RF (1964). Theoretical significance of dendritic trees for neuronal input–output relations. *Neural Theory and Modeling*.

[b56] Ribak CE, Nitsch R, Seress L (1990). Proportion of parvalbumin-positive basket cells in the GABAergic innervation of pyramidal and granule cells of the rat hippocampal formation. J Comp Neurol.

[b57] Ribak CE, Peterson GM (1991). Intragranular mossy fibers in rats and gerbils form synapses with the somata and proximal dendrites of basket cells in the dentate gyrus. Hippocampus.

[b58] Ruiz A, Walker MC, Fabian-Fine R, Kullmann DM (2004). Endogenous zinc inhibits GABA_A_ receptors in a hippocampal pathway. J Neurophysiol.

[b59] Sambandan S, Sauer JF, Vida I, Bartos M (2010). Associative plasticity at excitatory synapses facilitates recruitment of fast-spiking interneurons in the dentate gyrus. J Neurosci.

[b60] Sauer JF, Struber M, Bartos M (2012). Interneurons provide circuit-specific depolarization and hyperpolarization. J Neurosci.

[b61] Seress L, Gallyas F (2000). The use of a sodium tungstate developer markedly improves the electron microscopic localization of zinc by the Timm method. J Neurosci Methods.

[b62] Sik A, Ylinen A, Penttonen M, Buzsaki G (1994). Inhibitory CA1–CA3–hilar region feedback in the hippocampus. Science.

[b63] Smart TG, Hosie AM, Miller PS (2004). Zn^2+^ ions: modulators of excitatory and inhibitory synaptic activity. Neuroscientist.

[b64] Smart TG, Moss SJ, Xie X, Huganir RL (1991). GABA_A_ receptors are differentially sensitive to zinc: dependence on subunit composition. Br J Pharmacol.

[b65] Soltesz I, Mody I (1994). Patch-clamp recordings reveal powerful GABAergic inhibition in dentate hilar neurons. J Neurosci.

[b66] Spiridon M, Kamm D, Billups B, Mobbs P, Attwell D (1998). Modulation by zinc of the glutamate transporters in glial cells and cones isolated from the tiger salamander retina. J Physiol.

[b67] Sun HS, Hui K, Lee DW, Feng ZP (2007). Zn^2+^ sensitivity of high- and low-voltage activated Ca^2+^ channels. Biophys J.

[b68] Szabadics J, Soltesz I (2009). Functional specificity of mossy fibre innervation of GABAergic cells in the hippocampus. J Neurosci.

[b69] Talley EM, Cribbs LL, Lee JH, Daud A, Perez-Reyes E, Bayliss DA (1999). Differential distribution of three members of a gene family encoding low voltage-activated (T-type) Ca^2+^ channels. J Neurosci.

[b70] Tang AH, Karson MA, Nagode DA, McIntosh JM, Uebele VN, Renger JJ, Klugmann M, Milner TA, Alger BE (2011). Nerve terminal nicotinic acetylcholine receptors initiate quantal GABA release from perisomatic interneurons by activating axonal T-type (Ca_v_3) Ca^2+^ channels and Ca^2+^ release from stores. J Neurosci.

[b71] Toth K (2011). Zinc in neurotransmission. Annu Rev Nutr.

[b72] Traboulsie A, Chemin J, Chevalier M, Quignard JF, Nargeot J, Lory P (2007). Subunit-specific modulation of T-type Ca^2+^ channels by zinc. J Physiol.

[b73] Tsuda M, Imaizumi K, Katayama T, Kitagawa K, Wanaka A, Tohyama M, Takagi T (1997). Expression of zinc transporter gene, ZnT-1, is induced after transient forebrain ischemia in the gerbil. J Neurosci.

[b74] Veran J, Kumar J, Pinheiro PS, Athane A, Mayer ML, Perrais D, Mulle C (2012). Zinc potentiates GluK3 glutamate receptor function by stabilizing the ligand binding domain dimer interface. Neuron.

[b75] Vinet J, Sik A (2006). Expression pattern of voltage-dependent Ca^2+^ channel subunits in hippocampal inhibitory neurons in mice. Neuroscience.

[b76] Vogt K, Mellor J, Tong G, Nicoll R (2000). The actions of synaptically released zinc at hippocampal mossy fibre synapses. Neuron.

[b77] Weisskopf MG, Zalutsky RA, Nicoll RA (1993). The opioid peptide dynorphin mediates heterosynaptic depression of hippocampal mossy fibre synapses and modulates long-term potentiation. Nature.

[b78] Wu SM, Qiao X, Noebels JL, Yang XL (1993). Localization and modulatory actions of zinc in vertebrate retina. Vision research.

